# Risk Factors Associated with Pyogenic Spinal Infections among Intravenous Drug Users and Nonusers

**DOI:** 10.1155/2024/9938159

**Published:** 2024-07-26

**Authors:** Vivek K. Bilolikar, Brendan Gleason, Lee Kripke, Robert Merrill, Colin Whitaker, Jon Handal

**Affiliations:** Thomas Jefferson University Jefferson Einstein Hospital, Philadelphia, PA, USA

## Abstract

**Purpose:**

To identify the differences in patient factors, presentation, treatment course, and outcomes of intravenous drug users (IVDA) vs. nonusers (non-IVDA) presenting with pyogenic spinal infections. *Study Design/Setting*. Retrospective case series.

**Methods:**

We identified all cases involving spinal infections at our institution between May 2017 and January 2023. Postsurgical infections were excluded, and patients were separated into IVDA and non-IVDA groups. The patient charts were reviewed and analyzed for statistical or clinically significant differences using RStudio (2019 version 3.6.2). Our institutional review board approved this study, IRB# 2020-277, iRISID-2023-1384.

**Results:**

Fifty patients (29 males and 21 females) with primary pyogenic spinal infections were included in our study. There were fourteen patients (28.0%) in the IVDA group. The mean age in the IVDA group was 50.6 vs. 61.9 years (*p* < 0.05) in the non-IVDA group. The average length of stay (LOS) in the IVDA group was 15.8 vs. 14.0 days (*p* = 0.54) in the non-IVDA group, with no significant difference in readmissions or disposition. Twenty-three non-IVDA patients were diagnosed with diabetes, while eight IVDA patients had a psychiatric diagnosis (other than substance abuse). There were no significant differences in microbial isolate or the duration of antibiotics.

**Conclusion:**

In our study population, there is a high incidence of intravenous drug abuse (IVDA), psychiatric disease, diabetes, and chronic kidney disease. Analysis shows a trend of two distinct patient populations. Patients without a history of IVDA were significantly older than those with IVDA and significantly more likely to have medical comorbidities including hyperlipidemia, diabetes, chronic kidney disease, and malignancy than those with IVDA history. Patients with IVDA were younger with significantly higher rates of smoking and psychiatric disorders. IVDA patients struggled to receive continued psychiatric/addiction treatment after discharge, an area for significant improvement. Due to a small sample size and single urban institution setting, this study may be underpowered to demonstrate differences in healthcare resource consumption.

## 1. Introduction

Primary spinal infections represent a significant and complex clinical problem, exerting a substantial financial burden on an already strained healthcare industry. Emerging research has shown a troubling surge in primary pyogenic spine infections [1–3]. The trend may be attributed to two interrelated factors: (1) the widespread opioid epidemic and (2) the aging population with a higher prevalence of multiple medical comorbidities. Consequently, healthcare providers face the daunting task of managing more spinal infections in more vulnerable patients.

Pyogenic spine infections have a reported incidence of 0.2 to 2 cases per 100,000 per year [4, 5]. The infection is typically unimicrobial, with *S. aureus* accounting for 60% of infections and *Enterobacter* accounting for 30% [5]. The most common initial sources are infections in the genitourinary tract, lungs, or skin; the bacteria generally spread to the spine via hematogenous dissemination. Risk factors documented in the literature include diabetes mellitus; other chronic diseases, such as renal failure and cirrhosis; immunosuppressed states; and IV drug use [3, 5, 6].

Among the various subgroups of patients affected by pyogenic spinal infections, those who abuse intravenous drugs present an incredibly complex medical and social challenge. These individuals require a unique approach to their care, as their condition is intertwined with their drug abuse patterns and associated social determinants of health. Addressing their healthcare needs involves a comprehensive and multidimensional strategy encompassing medical interventions, specialized psychiatric evaluation, and prolonged hospitalization/institutionalization [7, 8]. IVDA patients are also at higher risks for developing/contracting immunocompromising coinfections like HIV/AIDS, hepatitis B, and hepatitis C [9]. Patients with pyogenic spinal infections who abuse intravenous drugs have a propensity for poor follow-up and continued drug abuse [1, 2]. Despite receiving medical attention and undergoing treatment, many individuals in this population struggle to break free from the grip of addiction [10], further complicating the management of their infections. It is crucial to recognize that effectively managing their spinal infection is intimately linked with addressing the underlying addiction issues, providing ongoing support, and facilitating access to addiction rehabilitation resources. The aim is not only to treat the infection but also to safeguard these patients and promote a healthier lifestyle.

Given the multifaceted nature of spinal infections in patients with a history of IVDA, healthcare providers must adopt a proactive and collaborative approach. Interdisciplinary teams comprising physicians, surgeons, addiction specialists, psychiatrists, social workers, and other healthcare professionals must work together to develop personalized treatment plans encompassing medical, psychological, and social interventions [8]. By incorporating harm reduction strategies, psychological counseling, addiction rehabilitation programs, and community support services, healthcare providers can maximize the chances of successful recovery and improve overall patient outcomes.

This study aims to identify the differences in patient factors, presentation, treatment course, and outcomes of intravenous drug abusers vs. nonabusers presenting with pyogenic spinal infections. This information can improve how we treat patients presenting with pyogenic spine infections. By recognizing the multifaceted nature of their condition, adopting a comprehensive approach that addresses their medical, psychological, and social needs, and fostering a supportive and understanding society, healthcare providers can strive towards improved outcomes and a brighter future for these patients.

## 2. Materials and Methods

### 2.1. Study Design

A retrospective chart review study was conducted to assess the clinical characteristics, treatment patterns, and outcomes of patients diagnosed with primary spinal infections. This study utilized deidentified medical records from Einstein Medical Center Philadelphia from May 1, 2017, to January 31, 2023. The Institutional Review Board (IRB) (#2020-277 and iRISID-2023-1384) reviewed and approved the study protocol.

### 2.2. Data Collection

A comprehensive chart review identified eligible patient records within the electronic health record system of the institution. Inclusion criteria encompassed patients of all ages with a documented primary pyogenic spinal infection diagnosis. Cases of secondary spinal infections, such as those resulting from postoperative complications, were excluded from the analysis. The patients were then grouped based on whether or not they actively used intravenous drugs (IVDA) (non-IVDA).

A standardized data collection form was developed and used. Three independent reviewers extracted the following variables from the patient medical records: demographic characteristics (e.g., age and gender), medical comorbidities (e.g., diabetes and malignancy), route of infection acquisition (e.g., intravenous drug abuse), laboratory findings (e.g., complete blood count and inflammatory markers), diagnostic imaging results (e.g., MRI and CT scan), microbiological data (e.g., culture results), treatment modalities (e.g., antibiotic therapy and surgical intervention), length of hospital and rehab stay, complications, and outcomes (e.g., resolution of infection and mortality).

The study adhered to strict ethical guidelines protecting patient privacy and confidentiality. All patient data were deidentified and our IRB granted a waiver of informed consent due to the study's retrospective nature and the minimal risk to patient privacy.

### 2.3. Data Analysis

Descriptive statistical analysis was performed to summarize the collected data. Continuous variables were presented as means with standard deviations, depending on their distribution. Categorical variables were summarized as frequencies and percentages. Subgroup analyses explored potential associations between patient characteristics and treatment outcomes. Statistical tests, such as Fisher-exact tests for categorical variables and *t*-tests for continuous variables, and 2-way ANOVAs for multigroup/variate analysis, were used where appropriate. A *p* value of less than 0.05 was considered statistically significant.

All data analysis was performed using RStudio (2019 version 3.6.2), and the results were consolidated and anonymized to maintain patient confidentiality.

## 3. Results

304 unique patients were identified via ICD9 and ICD10 coding query for spine infections (e.g., G06.1, G06.2, M46.22, M46.24, M46.26, M46.32, M46.33, M46.40, M46.44, M46.46) were treated at our institution between May 2017 through January 2023. Following manual review, excluding secondary, postsurgical, and nonpyogenic infections, 50 patients with primary pyogenic spinal infection were included in our analysis. Fourteen patients (28.0%) with a history of IVDA were identified. In total, there were 29 males and 21 females. The mean age in the IVDA group was 50.6 years (range: 30–75 years), and the mean age in the non-IVDA group was 61.9 years (range: 45–89 years), and the difference was statistically and clinically significant. Demographics are presented in Table 1. Patient comorbidities are summarized in Table 2. Of note, 23 (63.9%) patients in the non-IVDA group were diagnosed with diabetes, while eight patients in the IVDA group had a psychiatric diagnosis (in addition to substance abuse), both statistically significant. 30.6% of non-IVDA patients presented with chronic renal disease compared to 7.1% of IVDA patients (*p* = 0.032). 27.8% of patients in the non-IVDA group had alcoholic liver cirrhosis, and six of those ten patients also had chronic hepatitis C. This is compared to the IVDA group, where 14.1% of patients had liver cirrhosis secondary to chronic alcohol abuse and 35.7% had chronic hepatitis C without cirrhosis. In our study population, these differences were not statistically significant. We also noted that patients in the non-IVDA group were more likely to have a history of malignancy (16.7% vs. 0%, *p* = 0.012). Patients in the non-IVDA group had additional comorbidities, 4.0 vs. 3.7. Additionally, in our study population, the overall smoking rate was 74.0% and the difference between the IVDA and non-IVDA groups was statistically significant (92.8% vs. 66.7 *p* = 0.018). Among IVDA patients, 78.9% were diagnosed with bacteremia compared to 61.1% of non-IVDA patients, and the rate of endocarditis was 21.4% in IVDA patients and 2.8% in the non-IVDA group; neither of these differences was statistically significant within our study population. 11 patients (22%) had a documented psychiatric diagnosis. Three (6%) patients without IVDA had a documented psychiatric diagnosis while 8 (16%) patients with IVDA had a documented psychiatric diagnosis. Diagnoses are outlined in Table 3.

Table 4 summarizes the affected spinal regions that patients presented with; the most common region affected was the lumbar spine, followed by the thoracic and cervical regions. Two-way ANOVA demonstrated no statistically significant correlations between the spinal region and IV drug use. No significant differences or correlations existed between the type/number of imaging series and the affected spinal region and IVDA status.

Neurologic status on admission is summarized in Table 5 stratified by severity of neurologic deficit and IVDA status. 18% of patients did not have a formal neurologic exam documented on admission due to lack of cooperation or critical illness/intubation. 50% of patients did not have neurologic deficit. 20% of patients had mild motor deficits and 12% had severe motor deficit. Motor deficit was defined as mild if anti-gravity strength was preserved in all motor distributions and severe if there was inability to perform anti-gravity strength in any motor distribution.

Regarding treatment options, 92.8% of IVDA patients underwent surgical decompression for neural compression compared to 83% for non-IVDA. The difference was not statistically significant. Similarly, the number of levels decompressed was the same, 2.9. There was no difference in rate or type of instrumentation between groups, 35.7% vs. 25.8% (IVDA vs. non-IVDA, respectively)—all patients who received instrumentation received titanium implants.

A total of 5 (3 IVDA and 2 non-IVDA) patients received surgical intervention including anterior corpectomy. Each of these patients also underwent concomitant posterior decompression and instrumentation. The remaining 45 patients underwent posterior approach alone. Posterior surgery was performed via standard midline posterior approach to the cervical, thoracic, or lumbar spine as necessary.

At the time of surgery, cultures were taken via swab or biopsy. The patients who did not undergo surgery underwent a CT-guided biopsy by the interventional radiology team, which was then sent for culture. The isolated organisms are summarized in Table 6, and the differences noted are not statistically significant. In 7 cases (21.2%), methicillin-sensitive *Staphylococcus aureus* (MSSA) grew in the culture media, while methicillin-resistant *S. aureus* (MRSA) grew in 12 (36.3%). Other microbial isolates included *Burkholderia* [1], *Candida* [1], *Citrobacter* [1], *Klebsiella* [1], *P. acnes* [1], *Pseudomonas* [1], coagulase-negative staph [2], and *Proteus* [2]. Two patients, both non-IVDA, presented with multi-microbial infections. The average length of stay (LOS) in the IVDA group was 13.1 days, and the average LOS in the non-IVDA group was 15.8 days (see Table 7). There was no significant difference in readmission rates nor discharge to a rehab/SNF. All patients, except for one non-IVDA patient, were discharged with a prescription for extended antibiotics for at least 21 days. The antibiotic treatments were recommended by the infectious disease consultants and tailored to the culture results. 73.9% of non-IVDA patients were sent with a PICC line and IV antibiotics compared to 80% of IVDA patients (*p* = 1). It should be noted that there was significant variability within the non-IVDA group regarding LOS and duration of antibiotics. In our case series, there were three mortalities. Three patients required a reoperation, one for acute repeat irrigation and debridement, one for superficial wound dehiscence, and one for persistent deep wound infection.

Overall rate of follow-up was poor for patients in this study. All patients were advised to follow up outpatient with orthopedic spine team and infectious disease. Follow-up is outlined in Table 8. Less than half (42%) of patients with IVDA followed up with orthopedics, and 14% of IVDA patients followed up with infectious disease. 26% of patients without IVDA followed up with orthopedics and 17% followed up with infectious disease. All patients who were seen in the outpatient setting had a complete/intact neurological recovery at one-year follow-up. All IVDA patients were referred to drug cessation programs; however, none successfully registered with programs affiliated with our institution or partners.

## 4. Discussion

The findings revealed that two distinct groups of patients are diagnosed with pyogenic spinal infections. The IVDA group consists of younger patients who have fewer comorbidities, and the non-IVDA group consists of older patients who tend to have multiple comorbidities, the most common being diabetes mellitus followed by severe chronic renal disease. Our study identified age over 60 as a risk factor for pyogenic spinal infection. This is likely contributing to patients' overall immunocompromised state as numerous population-based studies have demonstrated that as we age, we compile additional comorbid conditions and risk factors, which would inhibit the full function of our immune system.

The ultimate treatment algorithm for both groups remained the same at our institution, with most patients in both groups undergoing surgical decompression of the affected spinal levels in the setting of neurological compromise. This is noted to be similar protocols to other similar studies in this field [11]. Postoperative treatment for both groups consists of extended antibiotic administration tailored to the isolated microbe, which is was determined and managed by our department of infectious disease.

The results are consistent with previous studies that have reported similar differences between the two populations. The prospective study conducted by Wang et al. [4] of 102 patients (51 of which were IVDA) demonstrated that the IVDA patients were, on average, 13 years younger (43 vs. 56). They also demonstrated a high rate of immunocompromising coinfections, where 45% had HIV, 25% had hepatitis B, and 84% had hepatitis C [4]. While this was not demonstrated in our study, any immunocompromise significantly increases the risk that patients may develop infections; more specifically, it increases their risk of infection with typically nonpathogenic or atypical microbes [9, 12]. Our study is consistent with the current literature regarding bacteremia, endocarditis, and pyogenic spinal infections. The current literature shows that approximately 75–90% of patients presenting with pyogenic spinal infections had culture-positive bacteremia [4, 5, 13]. However, the rate of endocarditis described in the existing literature is much more variable, as experts contend that this variability is likely due to an underdiagnosing of infective endo-vasculitis as the persistent infectious nidus as opposed to the traditional thinking that infective endocarditic vegetations may result in septic emboli, which ultimately cause diffuse/distant infections.

Our identified rate of *Staphylococcus* (MRSA and MSSA) infection is consistent with prior studies [3, 8, 13]. MRSA infection rates remain high in compromised populations (IVDU and medically complex patients). MRSA rates (40–60%) remain high in urban and crowded counties including Philadelphia [14, 15], with the rate of community-acquired MRSA increasing annually since 2009 [16].

Our study is consistent with existing literature demonstrating that diabetes mellitus is a significant risk factor for developing a pyogenic spinal infection [4, 6, 17]. While diabetes itself does not directly cause immunocompromise, poorly controlled diabetes makes patients more susceptible to infections primarily through impaired leukocyte and neutrophil function, impaired wound healing, and frequent dermal disruptions (fingerstick glucose monitoring and insulin injection) [18]. While our study did not identify statistically significant differences between our study groups regarding the prevalence of liver cirrhosis and hepatitis C infection, these conditions represent significant risk factors for patients to develop infections secondary to generalized immunocompromise. Liver cirrhosis causes (1) impaired protein synthesis, including immunoglobulins, (2) impaired phagocytosis and lymphocyte dysfunction, (3) chronic inflammation, and (4) general malaise/failure to thrive/malnutrition [19, 20]. While typically not considered an immunosuppressive condition, hepatitis C can also reduce the effectiveness of the immune system by altering the acute immune response through (1) direct complement inhibition, (2) chronic inflammation, and (3) general malaise/failure to thrive/malnutrition [21]. Additionally CKD can cause immunocompromise through several different pathways; the most notable are (1) uremia, (2) proteinuria of essential immune proteins/signaling molecules, (3) chronic inflammation, and (4) general malaise/failure to thrive/malnutrition [22]. Universally, these conditions can cause malnutrition and failure to thrive, a well-established risk factor for developing infections and further decompensation. Frequently less discussed, chronic inflammation significantly affects patients' immune systems; most notably, it leads to the upregulation of anti-inflammatory cytokines and the downregulation of proinflammatory cytokines, suppressing the acute immune response [23]. We must recognize these comorbid conditions to ensure patients are as medically optimized as possible before surgery and follow their procedures to minimize their risk for reinfection or recurrence.

In our study, instrumentation was used based on intraoperative decision making by attending surgeon regarding expected instability due to amount of bony decompression required. Any patient who received instrumentation received titanium instrumentation with good outcomes and no reoperations for further infection or implant failure. This is consistent with the current literature, demonstrating that titanium spinal implants are safe and reasonable options for use in an actively infected surgical bed [24–27]. Biological studies have demonstrated that *S. aureus* is less likely to form a biofilm on titanium than stainless steel, with tantalum alloys having even less susceptibility to film creation. This may contribute to the lower infection rate with titanium implants compared to stainless steel in primary and revision spine procedures [27].

This study highlighted an area for improvement at our institution, but more broadly, it applies to all healthcare providers and systems that care for patients who struggle with substance addiction. While all IVDA patients were referred to outpatient psychiatric and drug cessation programs, none were successfully enrolled within 90 days of discharge, which is not always in the physician's control. Attention and effort must be placed on effective posthospital addiction treatment and support. This represents the difficulty of system-based practice in treating IVDA and addiction. Pyogenic spinal infection is a symptom of the more extensive disease of addiction in the IVDA population. Drug cessation and addiction support are part of the treatment plan to help this group of patients in the long term. We recommend that a patient with a history of IVDA be considered for an in-patient consult for psychiatric evaluation and social services with planned transition to outpatient resources as appropriate.

This study has several limitations inherent to its retrospective design. Firstly, the data collected relied solely on the information documented in the medical records, which may be subject to variability and incomplete documentation. Secondly, the study was conducted in a single healthcare institution, which may limit the generalizability of the findings to other settings. Lastly, as a retrospective study, it is susceptible to selection bias and confounding factors.

## Figures and Tables

**Table 1:** Comparison of demographics between the IVDA group and non-IVDA group.

	Non-IVDA	IVDA	*p*
*N*	36	14	
Gender (F/M)	15/21 (41.6/58.4)	6/8 (42.9/57.1)	0.941
Age (years)	61.9 (45–89)	50.6 (30–75)	0.012

Parentheses indicate the percentage for categorical variables. Continuous variables are presented as mean with the range in brackets.

**Table 2:** Comparison of prevalence of comorbidities between the IVDA group and non-IVDA group.

	Non-IVDA (*N* = 36)	IVDA (*N* = 14)	*p*
Diabetes	23 (63.9)	1 (7.1)	<0.001
Kidney dysfunction	11 (30.6)	1 (7.1)	0.032
HIV	1 (3.8)	0 (0.0)	0.324
Hepatitis	8 (22.2)	5 (35.7)	0.215
Cirrhosis	10 (27.8)	2 (14.3)	0.145
Lung disease	5 (13.9)	4 (11.1)	0.302
Smoking	24 (66.7)	13 (93.8)	0.019
Malignancy	6 (16.7)	0 (0.0)	0.012
Rheumatoid	1 (2.8)	0 (0.0)	0.324
Cardiovascular disease	13 (36.1)	3 (21.4)	0.43
HLD	14 (38.9)	1 (7.1)	0.005
Hypothyroidism	3 (8.3)	0 (0.0)	0.083
Psychiatric disorder	3 (8.3)	8 (57.1)	0.004
Bacteremia	22 (61.1)	11 (78.6)	0.225
Endocarditis	1 (2.8)	3 (21.4)	0.133

Parentheses indicate the percentage for categorical variables.

**Table 3:** Psychiatric comorbidities.

Patient	IVDA	Psychiatric diagnosis
1	N	Schizophrenia, schizoaffective
2	N	Substance use disorder, bipolar, borderline
3	N	Anxiety, depression, alcohol abuse
4	Y	Substance use disorder, schizophrenia
5	Y	Substance use disorder, depression
6	Y	Substance use disorder, depression
7	Y	Substance use disorder, depression, schizophrenia
8	Y	Substance use disorder, anxiety, PTSD, bipolar
9	Y	Substance use disorder, depression, bipolar
10	Y	Substance use disorder
11	Y	Substance use disorder

**Table 4:** Comparison of infection location by spinal region between the IVDA group and non-IVDA group.

	Non-IVDA	IVDA
Cervical	3 (8.3)	2 (14.3)
Thoracic	13 (36.1)	6 (42.9)
Lumbar	20 (55.6)	6 (42.9)

Two-way ANOVA—no statistical difference. Parentheses indicate the percentage for categorical variables.

**Table 5:** Neurologic exam on admission.

Neuro exam	Number of patients	Non-IVDA (*N* = 36)	IVDA (*N* = 14)
No deficit	25 (50.0%)	14 (28.0%)	11
Sensory only	0 (0)	0 (0.0%)	0 (0.0%)
Mild motor	10 (20.0%)	10 (20.0%)	0 (0.0%)
Severe motor	6 (12.0%)	3 (6.0%)	3 (6.0%)
Not recorded	9 (18.0%)	9 (18.0%)	0 (0.0%)

Examination is categorized as no deficit, sensory deficit only, mild motor deficit (anti-gravity strength preserved in all motor groups), and severe motor deficit (at least one motor group with less than anti-gravity strength). 9 patients did not have a formal neurological examination recorded on admission due to lack of cooperation and critical illness/intubation. *χ*^2^*p*=0.10.

**Table 6:** Comparison of organisms identified from biopsies between the IVDA group and non-IVDA group.

	Non-IVDA (*N* = 36)	IVDA (*N* = 14)	*p*
Organism			0.126
MRSA	9 (25.0)	4 (28.6)	
MSSA	12 (33.3)	6 (42.9)	
Coag-negative staph	4 (11.1)	0 (0.0)	
*Burkholderia*	0 (0.0)	1 (7.1)	
*Candida* ssp.	1 (2.8)	0 (0.0)	
*Citrobacter koseri*	1 (2.8)	0 (0.0)	
*Klebsiella*	1 (2.8)	0 (0.0)	
*P. acnes*	0 (0.0)	1 (7.1)	
*Proteus*	2 (5.6)	0 (0.0)	
*Pseudomonas*	0 (0.0)	1 (7.1)	
Other Gram-negative	4 (11.1)	0 (0.0)	
Negative cultures	3 (13.0)	1 (7.1)	

Parentheses indicate the percentage for categorical variables.

**Table 7:** Comparison of posthospital treatment plan.

	Non-IVDA (*N* = 36)	IVDA (*N* = 14)	*p*
PICC line	31 (86.1)	10 (71.4)	0.301
Readmission	22 (61.1)	6 (42.9)	0.266
Disposition			0.94
Home	10 (27.8)	4 (28.6)	
Other	4 (11.1)	2 (20.0)	
Rehab	22 (61.1)	8 (57.1)	
Length of stay (days)	15.8 (11.8)	13.1 (8.6)	0.300
Antibiotic duration (days)	46.0 (38.3)	38.1 (9.2)	0.258

Categorical variables are presented with counts and the percentage of the total. Continuous variables are presented with the mean and standard deviation.

**Table 8:** Outpatient follow-up.

	Non-IVDA	IVDA
Ortho follow-up	13 (26)	6 (42)
ID follow-up	6 (17)	2 (14)

All patients were advised to follow up outpatient with their operative orthopedic spine surgeon and infectious disease. Chi-squared *p*=0.73.

## Data Availability

The anonymized chart review data used to support the findings of this study are available from the corresponding author upon request.
